# Diarsenol in the Treatment of Syphilis: Clinical Report

**Published:** 1916-01

**Authors:** E. P. Merritt

**Affiliations:** Atlanta, Ga.


### DIARSENOL IN THE TREATMENT OF SYPHILIS: CLINICAL REPORT.

By E. P. Merritt, M. D., Atlanta, Ga.

To know the real virtue of any one thing, referring especially to medicine, it takes time, labor and experience, before we have a thorough and true knowledge that we may depend upon. It is very easy to think we are right, but ofttimes we do not know we are right.

My object in discussing Diarsenol and presenting these case histories is for one reason and that is that we may get a discussion by the profession who have used this preparation and thereby gain valuable information that we may all derive benefit therefrom. If it has the therapeutic value equal to that of Salvarsan, the sooner we know it the better; if it has not, we want to know that also. If it is not the same thing, surely it is a very good imitation, and let us hope it is as valuable. Time and experience alone will tell the tale.

It is mixed and given the same as Salvarsan. The odor is somewhat stronger and more garlic-like. The reaction or toxicity, if you please, equals that of Salvarsan, but no more, has been my experience.

I have administered it to eleven different patients. This number being entirely too small to come to any definite conclusion upon, therefore, it will be classed as preliminary.

Six cases of Tertiary Syphilis.

Five cases of Primary Syphilis.

In four of the six Tertiary cases mentioned, was unable to tell whether they were benefitted or not, as there were no lesions to be seen, but in two there was marked improvement. One patient with Syphilitic Alopecia plus Syphilides of the Scalp, showed marked improvement. The sores disappeared in four days, and constitutional symptoms were relieved. The other case was Sirpiginous ulceration of buttocks, which showed marked improvement in five days. None of these cases had any reaction from the injections worthy of mention:

The five primary cases were as follows:

Case 1. Came to me December 4, 1915. Sore on penis—duration about five weeks. Had been taking Mercury and Pot. Iod. for two weeks previous without any marked results. Sore examined and found to be typical Chancre. The inguinal glands were typical of Syphilis. Was advised to take at once Diarsenol, which he took 0.4 GM December 6th and in seven days the sore was healed over as you see. Note the following:

The Mercury and Iodide did not seem to influence the sore very fast and I attribute the rapid healing to Diarsenol—in this case. There was practically no reaction from this dose.

Case No. 2. Came to me October 24, 1915. Examination showed very small insignificant sore on the under border of penis, which was treated by me for a few days. It did not heal very rapidly, and about this time the patient left the City, and returned December 28th, with very extensive area involved and the secondary eruption very faint. His blood was tested on his request and found xxxx, as it was a very plain, clean-cut case of syphilis. He had the following symptoms, which are worth remembering: Frontal headache, Inguinal Glands enlarged in masses, Epitrochelear Glands enlarged, very nervous, insomnia, loss of appetite and loss in weight. I gave him December 30th 0.3 gm Diarsenol. The next day he felt very much relieved of all his symptoms and in three days was entirely relieved. The sore healed in ten days. The reaction from this dose was as follows: The night following the injection—chills,—diarrhoea —nausea—headache. The sore, he says, burned and hurt all night. This case was Secondary Spyhilis instead of Primary.

Case No. 3. Patient presented himself for treatment December 28th, with sore on penis, which looked very suspicious of Syphilis, sore had been there two weeks. Three or four examinations of the secretion and finally the Spirochete Pallada were found. Sore was about the size of a dime. On December 30th 0.3 gm Diarsenol injected introvenous and in 48 hours there was noticeable improvement and in five days the sore was entirely over. The patient had slight reaction but not severe.

Case No. 4. Patient presented himself for treatment November 11th, giving history of sore on penis for two weeks previous. Examination revealed sores all around the frenum and on the foreskin with severe phimosis. The Ducrey bacillus was found in secretion, also the Sprochete. Of course, this meant mixed infection. Patient was treated locally and given large doses K. I. and Hydg. Mercury inunction, etc.—could not obtain D. Patient gradually improved and was given 0.4 gm

Diarsenol December 11, but he was improving slowly beforehand and it being a mixed infection and so stubborn, I could not tell whether he derived much benefit from the dose or not, except after the administration of the Diarsenol, he said he felt very much relieved. The reaction from the dose was not noticeable.

Case No. 5. Came to me December 22nd, with sore on penis of three weeks' duration, which was a typical Chancre. Secretion from it showed Sprochete Pallida in numbers. December 23 he was given 0.5 gm Diarsenol and in three days the sore was rapidly healing and induration softening up and in eight days' time the sore was practically well. This patient did not take K. I. and Hydg. for two or three days after administration of the drug, but before I put him on it I could see a marked improvement. The reason I put him on K. I. and Hydg. was, I didn't want to take any chances, as his wife was due back in the City in a week. This patient had a mild reaction from this dose.

### Conclusion.

I do not say that this preparation is as good as Salvarsan, for I do not know, but watching the result, as I have in these cases, and considering the same results I would have obtained from Salversan under the same circumstances. I am compelled to believe there is no material difference, as far as the rapid healing of Syphilitic sores are concerned.

